# Rapid Clearing for High Resolution 3D Imaging of Ex Vivo Pancreatic Cancer Spheroids

**DOI:** 10.3390/ijms21207703

**Published:** 2020-10-18

**Authors:** Eliana Steinberg, Natalie Orehov, Katerina Tischenko, Ouri Schwob, Gideon Zamir, Ayala Hubert, Zakhariya Manevitch, Ofra Benny

**Affiliations:** 1 The Institute for Drug Research, The School of Pharmacy, Faculty of Medicine, The Hebrew University of Jerusalem, Jerusalem 91120, Israel; Eliana.steinberg@mail.huji.ac.il (E.S.); Natalia.orehov@mail.huji.ac.il (N.O.); Katerina.tischenko@mail.huji.ac.il (K.T.); Ouris@ekmd.huji.ac.il (O.S.); 2 Department of Surgery and Transplantation Unit, Hadassah-Hebrew University Medical Center, Ein Kerem, Jerusalem 91120, Israel; Gideonza@ekmd.huji.ac.il; 3 Sharett Institute of Oncology, Hadassah-Hebrew University Medical Center, Ein Kerem, Jerusalem 91120, Israel; Ayalah@hadassah.org.il; 4 The Core Research Facility, The Hebrew University of Jerusalem, Jerusalem 91120, Israel; Zakhariyam@ekmd.huji.ac.il

**Keywords:** spheroids, clearing, 3D imaging, pancreatic cancer

## Abstract

The currently accepted imaging methods have been a central hurdle to imaging the finer details of tumor behavior in three-dimensional (3D) ex vivo multicellular culture models. In our search for an improved way of imaging tumor behavior in its physiological-like niche, we developed a simple, efficient, and straightforward procedure using standard reagents and imaging equipment that significantly enhanced 3D imaging up to a ~200-micron depth. We tested its efficacy on pancreatic spheroids, prototypes of high-density tissues that are difficult to image. We found we could both save time with this method and extract information about pancreatic tumor spheroids that previously was difficult to obtain. We were able to discern clear differences in the organization of pancreatic tumor spheroids generated from different origins, suggesting cell-specific, inherent, bottom-up organization with a correlation to the level of malignancy. We also examined the dynamic changes in the spheroids at predetermined time points, providing important information related to tissue morphogenesis and its metabolic state. Lastly, this process enabled us to assess a drug vehicle’s potential to penetrate dense tumor tissue by improving our view of the inert particles’ diffusion in the 3D spheroid. This clearing method, a simple procedure, can open the door to more accurate imaging and reveal more about cancer behavior.

## 1. Introduction

Pancreatic ductal adenocarcinoma (PDAC) is one of the most fatal cancers worldwide, with a 5-year survival rate of less than 5% that has not substantially improved in the last decade. The poor prognosis results are due to the often-late diagnosis of the disease and early metastatic development, making only one-tenth of the patients suitable candidates for surgery [1,2]. Pancreatic adenocarcinoma is a very heterogeneous tissue comprised of many different stromal cell types, including fibroblasts, pancreatic stellate cells, and immune cells. This tumor is commonly characterized by a dense extracellular matrix (ECM) which includes collagen, fibronectin, and proteoglycans. The dense ECM creates a unique tissue architecture with an unusual development of blood and lymphatic vessels. In many cases, the pancreatic tumor is characterized by hypo-vascularity which, in combination with the stiff ECM, causes a low drug perfusion contributing to therapeutic resistance [3,4].

Due to the role of tissue composition on biodistribution in pancreatic tumors as well as other dense tissues, there is a great need to develop an ex vivo model that will properly mimic the physiologically unique microenvironment, thus providing biologically relevant information. Such an optimal model should maintain authentic key tissue features in order to be relevant for drug screenings. Standard cell culture assays are mostly based on monolayer growth due to their low cost and simplicity. This form of cell growth provides a valuable means for high-throughput screening of compounds. However, the complexity of spatial cell organization that exists in vivo is missing and thus is unable to recapitulate the elements of the tissue’s microenvironment [5,6]. In comparison, three-dimensional (3D) cellular models have higher physiological relevance since they possess spatial communication between cells [5], display cell interaction with extracellular matrix components, and experience nutrient and oxygen exchange, leading to a metabolic gradient [6,7] similar to those found in physiological tissues.

The technology supporting 3D cultures is rapidly advancing and is being used for basic biology, drug screening, tissue engineering, and more recently, for personalized medicine. There are many 3D techniques, each having a unique feature, such as: growing tissue specimens ex vivo [8], bioprinting cells in ECM [9], scaffold-based models [10], microfluidic devices [11], organoids [2], and multicellular tumor spheroids [12]. These models became important for cancer research and are used to study cancer pathogenesis, cell signaling, metabolism, metastasis propagation, and drug efficacy.

Due to sample thickness, two of the main challenges common to 3D ex vivo models are: visualization, and extracting valuable biological information related to the tissue’s state under different conditions. Information related to the spatial organization, biomarker distribution, and tissue viability in respect to the exact site in the tissue are commonly overlooked due to the difficulty of imaging thick samples, necessitating the use of bulk analysis. In most cases, only the sample volume or bulk viability is measured. Monitoring just the average values in spheroids misses the heterogeneity in the tissue-like environment. Indeed, typical biological tissue samples larger than several dozens of microns are not transparent, making it hard to image them using simple light microscopy. Accomplishing this is even more difficult when dealing with dense tissues like PDACs. Fluorescence imaging is optional, but not trivial; generally, the ability to achieve detailed imaging is limited by the thickness of the sample, depending on the microscope technology and the type of fluorophores used.

Solving the problem of optic limitation in dense tissues is critical for the study of drug delivery and tissue features. In that regard, it is well known that biomolecules or particulate drug carriers often exhibit a limited permeability in dense tumor tissues, including the pancreatic tumor. We, as well as others, have shown that spheroids can deposit dense ECM and thus be used ex vivo as a model for the pancreatic tumor [13,14,15,16]. Previous attempts to adapt optic clearing protocols for spheroids and organoids typically required either the use of light sheet microscopes, special flow chip devices, physical sectioning, or long procedures [17,18,19,20].

We now show that by using a straightforward and easily executed method for obtaining high resolution visualization of thick samples, important information regarding tissue morphogenesis and metabolism can be extracted. Confocal fluorescent images of pancreatic tumor spheroids, post-clearing process, are obtained at predetermined time points, and their dynamic spatial organization is detected in microscale diameters ranging from 200 to 500 microns. The clearing technique presented here requires minimal low-cost reagents suitable for standard confocal imaging that can be applied to analyze morphology and cellular interactions inside a given spheroid. We found that the cellular origin of the pancreatic tumor dictates not only the morphology and cell–cell adherence, but also interactions with stromal cells and the overall capability for particle penetration, possibly due to a differential ECM deposition. The biological insights and methodology can be further used for other types of cancers, including tissues with desmoplasia and fibrosis.

## 2. Results

Pancreatic cancer cell lines BxPC-3, AsPC-1, and PANC-1 were processed for imaging post-clearing procedure. All these cell lines were able to form stable and uniform spheroids after a relatively short period of 1–4 days in a 3D Petri dish array. Notably, BxPC-3 cells formed the most stable and compact spheroids as soon as after 1 day and were selected for further studies.

### 2.1. Spheroid Imaging with and without Clearing

To optimize the spheroid clearing method, we tested different concentrations and incubation times with Triton X-100 (0.01–0.2% from 15 min up to 24 h) for spheroid permeabilization. Experiments were performed qualitatively using 2–3 repeats. In addition, different clearing agents, including glucose, 100% glycerin, 85% glycerin, and RapiClear (SUNJin Lab, Taiwan) were used in order to modify sample refractive indexes. Long incubations with Triton X-100 or the use of high concentrations (0.2% as recommended by RapiClear protocol) led to a complete wash off of the fluorescent dyes. Glucose as a clearing agent, showed very limited results and did not improve the ability to perform deep imaging. RapiClear, 100% glycerin, and 85% glycerin provided similar results and enabled imaging of up to ~200-microns into the depth of the spheroid. Based on this, we selected 85% glycerin to be the most suitable and simple-to-use material due to its reduced viscosity in comparison to 100% glycerin and due to its lower cost compared with RapiClear.

**Figure 1:**
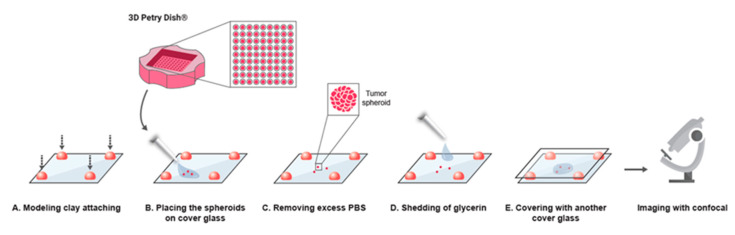
Illustration of the clearing procedure and the technical steps carried out prior to confocal imaging. In the first step, ~0.5 × 0.5 cm of modeling clay is attached on each corner of the cover glass and a drop of PBS containing 2–3 spheroids is gently spread in the center of the cover glass. The remaining PBS solution is removed and 50 µL of 85% glycerin is added on top of the spheroid sample. An additional cover glass is placed precisely and carefully on top and is then pressed slightly to allow ~1 mm space between the 2 cover glasses, enabling convenient and improved confocal imaging to be performed.

**Figure 2:**
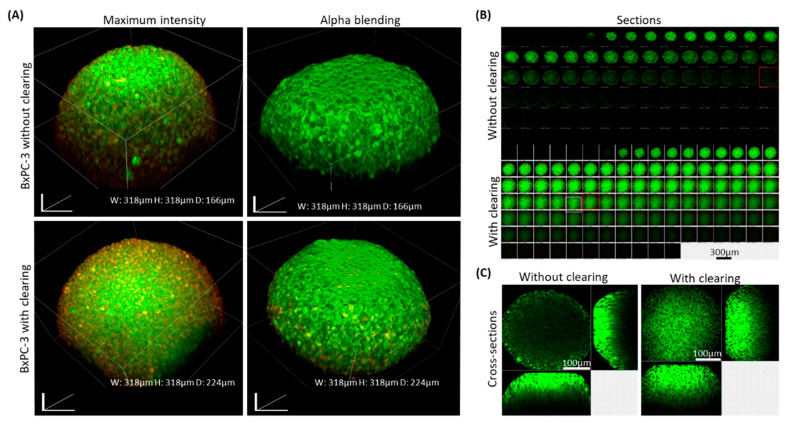
BxPC-3 spheroids with and without clearing treatment prior to imaging. BxPC-3 spheroids comprised of 10,000 cells were fixed and permeabilized 72 h after preparation. Spheroids were stained with Coumarin-6 (green) and DiD (red). (**A**) 3D projection imaging was performed with and without 85% glycerin clearing treatment prior to imaging. (**B**) Optic sections carried out in 2-micron steps of cleared and uncleared BxPC-3 spheroids. (**C**) Cross-sectioning of cleared and uncleared spheroids performed in 112 µm depth from the top.

Following the clearing process and sample preparation (as illustrated in Figure 1 and detailed in the Section 4), BxPC-3 spheroids were imaged in 3D projection mode in 2 variations: maximum projection mode and alpha-blending mode (Figure 2A). The cleared samples produced sharp images through the deep cell layers, showing details in the periphery and core.

Figure 2B shows the optic sections of selected spheroids carried out in 2-micron steps. As can be seen, the cleared samples remained vivid, sharp, and consists of comprehendible structures even at a depth of ~200 microns. In contrast, the uncleared sample shows blurry images already at ~50 microns depth, where only the edges are detected clearly. The internal section at the depth of 112 microns of cleared and uncleared samples (Figure 2C) shows the clear differences between these samples (the edges of the cleared spheroids appear blurrier compared with the uncleared spheroids; this is due to the core of the cleared samples being very bright, causing the edges to appear less clear).

### 2.2. Morphology and Spatial Organization of Spheroids Generated from Different Pancreatic Cancer Cell Origins

**Figure 3:**
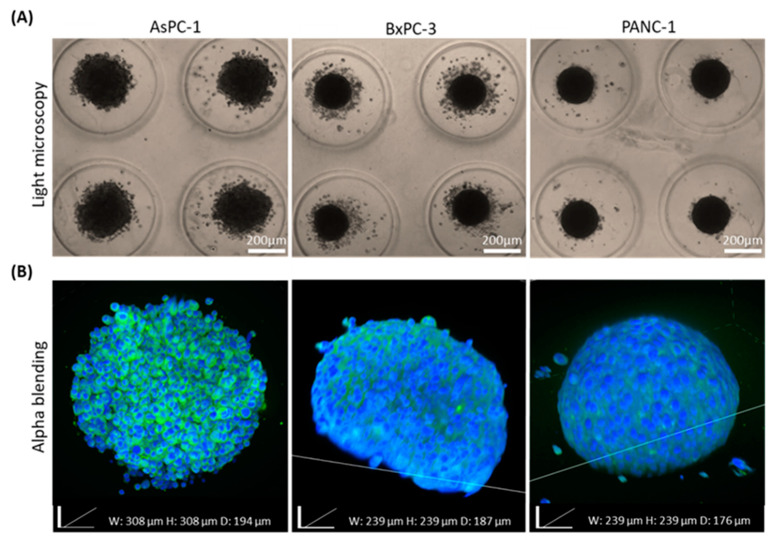
Morphological comparison of pancreatic adenocarcinoma spheroids generated from different origins. (**A**) Light microscopy bright field images of spheroids originated from different human pancreatic cancer cell lines (AsPC-1, BxPC-3, and PANC-1). (**B**) Corresponding fluorescence stack images of 3D spheroids stained with DAPI (blue) and Coumarin-6 (green) using confocal microscopy.

The architecture of spheroids, assembled from distinct pancreatic cancer cell lines, as well as their capacity to remain intact, were compared using the clearing process. Three types of human pancreatic cancer cell lines were used: AsPC-1, derived from pancreatic metastases found in the ascites, BxPC-3 and PANC-1, derived from primary tumors [21]. Spheroids were imaged with a light microscope before the clearing process, as shown in Figure 3A. While the majority of the AsPC-1 cells formed well-defined spheroids, it can be seen in Figure 3B that their outline surface is characterized by rough, sharp edges of cell aggregates.

In contrast, BxPC-3 cells assembled perfectly round-shaped spheroids with highly visible borders. However, in comparison with the other cell lines, many of its cells continued to grow outside the main spheroid body. PANC-1 spheroids resemble BxPC-3 spheroids by shape and diameter but as opposed to BxPC-3, contain fewer surrounding cells and, as a result, create more rounded spheroids. By comparing the confocal images to the light microscopy images, obtained prior to the clearing process, we found that the integrity of all the 3 spheroid types remained the same before and after the clearing process. Morphologically, BxPC-3 and PANC-1 spheroids preserved similarity regarding their roundness and smooth exterior with no projections, in contrast to AsPC-1 spheroids which formed a round, yet rougher, structure containing protrusions. To ensure that the clearing process did not affect the spheroids’ integrity and size, light microscopy images of BxPC-3 and AsPC-1 spheroids were taken and compared before and after each step of the clearing process, as can be seen in Figure S1.

### 2.3. Morphology Changes in BxPC-3 Spheroids Observed at Predetermined Time Points

**Figure 4:**
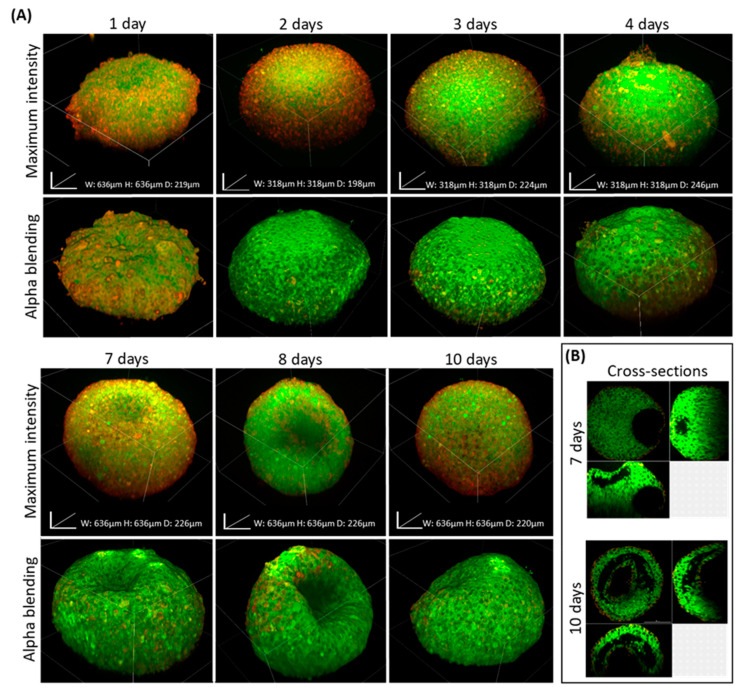
Dynamic 3D changes of BxPC-3 spheroids observed at predetermined time points. (**A**) Z-stacking images of BxPC-3 spheroids were reconstructed into 3D images portraying spheroids fixed at predetermined time points post-seeding: 1, 2, 3, 4, 7, 8, and 10 days and stained with Coumarin-6 (green) and DiD (red) (the day 3 image is the same cleared spheroid shown in Figure 2A). (**B**) Cross-sections of spheroids fixed at day 7 and 10, showing a retained outer structure with progressive hollowing of the core.

The dynamic nature of 3D grown samples includes cell–cell interactions, spatial assembly, and deposition of ECM components. Modification in metabolic nutrient exchange as a result of sample growth leads to hypoxia and eventually to necrosis over time. To follow spheroid organization at different time points, we monitored and observed spheroids over a 10-day period. Since spheroids are fixed prior to imaging, continuous observation over time was not possible. Samples were imaged by confocal microscopy as presented in Figure 4A on days 1, 2, 3, 4, 7, 8, and 10.

During the first 3 days, spheroids appeared intact, both from the outside and the inside, showing gradual growth without visible hollow areas. On day 4, a detectable breaching outer layer appeared, forming a small hole (~30 microns). On days 7 and 8, spheroids underwent considerable deformations and the inner site became hollow, leading to the collapse of the outer layer (Figure 4A,B). On day 10, only the outer layers of cells composing the spheroids retained their structure while the core was hollow. As shown in Figure 4B, the morphology changed into a flat “disk”-like structure. 

### 2.4. Cellular Organization of Stromal and Tumor Cells Co-Cultures Obtained from Different Origins

**Figure 5:**
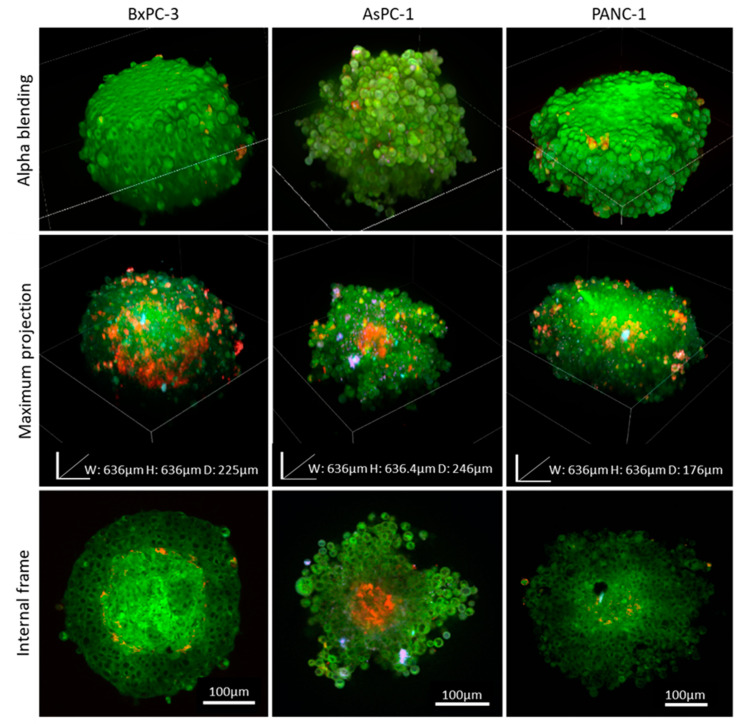
Co-cultured spheroids mimic the tumor microenvironment. Co-cultured spheroids comprised of 10,000 cells at a ratio of 6:2:2 of pancreatic cancer cells: patient-derived pancreatic cancer fibroblasts: HUVECs, respectively. Samples were fixed 4 days after seeding and immunolabeled with anti-CD31 (red), anti-fibroblasts (purple), and Coumarin-6 (green), and imaged post-clearing.

One of the key questions in cancer research is how the origin of cancer cells affects their interactions with stromal cells and whether they create distinct “tissue”-like organization. To extract biological data related to cell-cell crosstalk, we utilized the clearing method and analyzed hybrid co-culture spheroids consisting of 3 cell types: (1) pancreatic cancer cell lines obtained from 3 origins: BxPC-3, AsPC-1, and PANC-1 (6000 cells/spheroid), (2) patient-derived pancreatic cancer fibroblasts (2000 cells/spheroid), and (3) human umbilical vein endothelial cells (HUVECs) (2000 cells/spheroid). Samples were assembled into spheroids over a period of 4 days and then were imaged (Figure 5).

Immunofluorescence and imaging of the internal sections of the co-cultured spheroids were performed to detect the arrangement of the different tumor/stroma cell types post-self-assembly into spheroids. In these specific conditions, after 4 days, we detected only a few fibroblast cells, and almost all the spheroid samples were mainly comprised of cancer cells with a small percentage of endothelial cells. Clear differences were found in the spheroid morphology between the different cancer origins: BxPC-3 spheroids were the most compact, smooth, and spherical, AsPC-1 were irregular shaped with sprouting, and PANC-1 showed an intermediate phenotype. The most striking differences were in the formation of a fibrotic acellular core in PANC-1 and BxPC-3, while in AsPC-1, the core was populated with endothelial cells.

### 2.5. Interaction of Particles with BxPC-3 and PANC-1 Spheroids 

**Figure 6:**
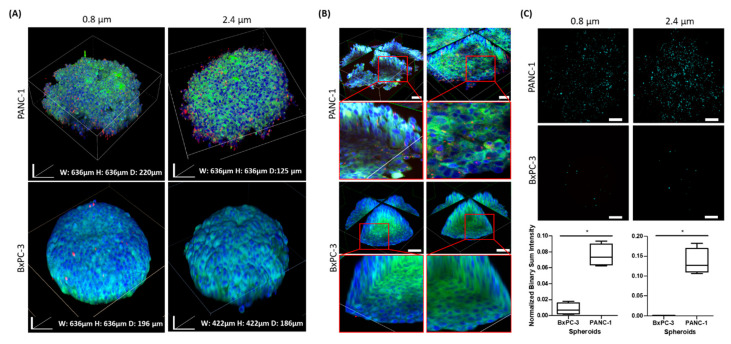
Particle penetration and biodistribution studies in BxPC-3 and PANC-1 spheroids as an ex vivo tumor model. BxPC-3 and PANC-1 spheroids were incubated with either 0.8 or 2.4 µm size fluorescent purple microparticles (red) over a period of 24 h. The spheroids were fixed, permeabilized, stained with DAPI (blue) and Coumarin-6 (green), and cleared. (**A**) Z-stacking images of BxPC-1 and PANC-1 spheroids were reconstructed into 3D images showing surface interaction with particles. (**B**) Optic cross-sections of the spheroids show interactions with particles in the deep cell layers. Scale bar = 50 µm. (**C**) Representative stack-projection images of 0.8 and 2.4 µm particles detected in BxPC-3 and PANC-1 spheroids after 24 h of incubation. The red color was converted to light blue to increase color contrast so as to enhance optical visualization. Graphs show quantification of the particles’ total projected fluorescent signals measured by NIS Elements AR Analysis software, *n* = 4, *p < 0.03. Scale bar = 100 µm.

To study the interactions of particles as a model for drug carriers in dense tissues, 2 micron-size inert particles (0.8 and 2.4 µm) were used with pancreatic cancer spheroids. Using the clearing method, we were able to compare spheroids comprised of cells with a high particle uptake capacity (PANC-1) to spheroids comprised of cells with a low uptake capacity (BxPC-3) [15]. Both spheroid types interacted with particles of 0.8 and 2.4 microns in diameter after 24 h of incubation. Figure 6A shows higher interactions of both particles with PANC-1 spheroids compared with BxPC-3 spheroids.

Figure 6B presents a central optic cross sectioning of the spheroid together with particles of both sizes, validating penetration of particles and not only superficial adherence on the surface of PANC-1 spheroids. Quantification of the fluorescent signal originated by the labeled particles demonstrated a significantly higher particle penetration in PANC-1 spheroids compared with BxPC-3 spheroids with the 2 particle diameters tested (Figure 6C). Unlike PANC-1, BxPC-3 showed minimal interaction with the particles, where only a small number of them were detected on the surface. 

### 2.6. Interaction of Particles with AsPC-1 Spheroids Post-Enzymatic Treatment

**Figure 7:**
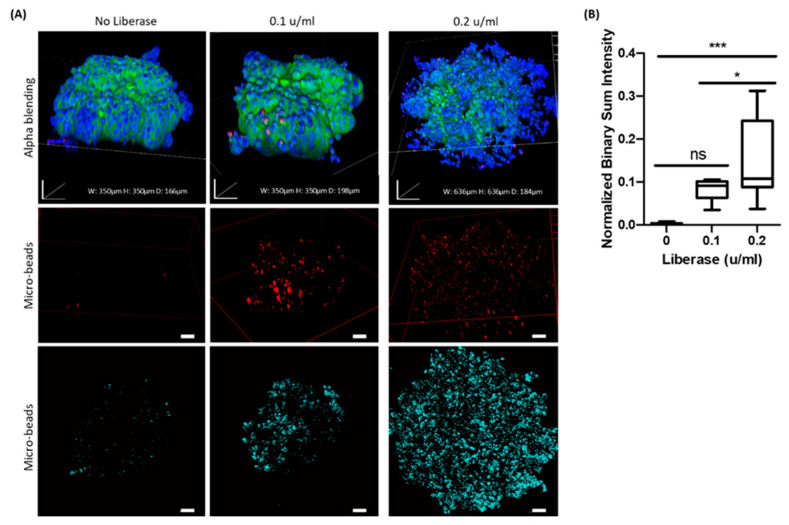
The effect of extracellular matrix “relaxation” of AsPC-1 spheroids as an ex vivo tumor model on particle diffusion. The penetration of 0.8 μm fluorescent purple microparticles into AsPC-1 spheroids treated with Liberase (0.1 or 0.2 of Wunsch units/mL) was compared with untreated spheroids. Spheroids were incubated with the particles (red) for 24 h and stained with DAPI (blue) and Coumarin-6 (green). (**A**) Alpha blending mode represents 3D stacking and reconstitution into a whole spheroid. Labeled particles demonstrated both adhesion and penetration into the spheroids’ inner layers, depending on the concentration of the enzymatic treatment. Internal optical sections demonstrated the presence of particles in the depth of the spheroid (80 µm). The red color of the particles observed in this figure was converted to light blue to increase contrast for visualization purposes. Scale bar = 50 µm. (**B**) The graph represents the quantification of 0.8 µm particles that penetrated the spheroids with or without Liberase treatment, *n* = 5, *p < 0.03, ***p < 0.0006, ns, not significant.

To further understand whether modification and “relaxation” of the dense cell aggregates by enzymes would improve the penetration and adhesion of the particles to the spheroids, we compared particle uptake in AsPC-1 spheroids with and without a short treatment with a mixture of collagenase and neutral protease enzymes that target collagen and non-collagen proteins that constitute the extracellular matrix. Two concentrations of enzymatic treatments were used, as described in the Section 4. Figure 7A demonstrates a pattern of particle interaction with 3 types of AsPC-1 spheroids (2 groups treated with different concentrations of enzyme and 1 group not treated) captured in different perspectives: Alpha blending images portray the 3D shape and size of each spheroid and micro-particle images depict in 3D the overall extent of particle interaction with the spheroids. Internal slices indicate the capacity of the particles to penetrate deeper compartments of the spheroids, and Figure 7B shows statistically different penetration in accordance with higher enzyme concentrations.

## 3. Discussion

Great efforts have been made to recapitulate the in vivo tumor microenvironment and the stromal niche ex vivo. Among the different cancer types, pancreatic adenocarcinoma is of particular interest due to its unique microenvironment, which includes activated fibroblasts and pancreatic stellate cells that form a highly dense desmoplastic tissue. The high ECM content results in a fibroblastic capsule that imposes a barrier to drug penetration, along with non-functional blood micro vessels that further reduce biodistribution in these tumors [22,23,24]. Given that spheroids acquire tissue-like features and exhibit heterogenic structure, bulk analysis or surface imaging is not always enough to extract important biologically relevant information. Disintegration of spheroids and the subsequent examination of suspended liberated cells is possible, although sometimes it is challenging due to entrapment in ECM or the stickiness of the sample. Moreover, in suspension, the important information related to spatial organization of the cells is lost, and only the composition or the cell ratio in the sample can be analyzed. 

For these reasons, it is essential that improved methods for detailed imaging of 3D samples be developed and it is desirable that they be compatible with standard laboratory equipment. Today’s most commonly used imaging techniques can only characterize cells that are present at the surface of the 3D cell model, thus providing an outline image of spheroids or other thick tissues. Since peripheral cells are the most exposed to oxygen and nutrients, they are not very representative of the entire cell population’s biology in a given sample. In fact, they frequently show a distinct biology compared with cells that reside in deeper layers of the 3D culture. Attempts to achieve better imaging and optical signals from deeper layers commonly require special microscopes, sectioning followed by histology, or fluorescent staining [25,26]. These methods are complex, time consuming, and generally disrupt the spheroid’s native structure, and are not always successful in providing enough cell detail and extracellular matrix structures inside the spheroid. Thus, there is a compelling need to develop a straightforward imaging technique of the entire spheroid, using standard equipment that could show different tissue-like features and provide data related to spatial heterogeneity.

Until now, significant efforts have been focused on improving optical imaging of thick native tissues through clearing agents, with an emphasis on maximizing fluorescent signal collection by reducing light scattering [27]. This strategy is mostly achieved through the same principle of matching the refractive indexes of different biologic tissue layers to the solvent by altering, discarding, and exchanging some of its constituents [27,28,29]. For example, with Sca*l*eS [30], the samples become mechanically fragile and are susceptible to breaking apart [27,31]. SeeDB [32] solution is highly viscous and difficult to manipulate, resulting in the loss of many samples. 3DISCO involves a step of tissue dehydration that causes sample shrinkage [29]. And lastly, ClearT2 [33] requires exposure to a high concentration of formamide and poses serious safety issues due to its teratogenicity [27,31].

Recently developed tissue clearing methods have been used to produce high quality images [25], but most of the commercially available protocols last about 72 h and are extremely expensive. Some studies attempted to adapt tissue clearing procedures for spheroid and organoid protocols [17,18,19,20], however, these studies used light sheet microscopy and not a standard confocal microscope, and they required long procedures or use of specific designated microchips. 

In the method presented in this paper, we significantly shortened the processing time from days to less than 2 h (including all processing steps) and substantially reduced the cost of the imaging by using standard reagents and equipment. We removed, to some degree, the lipid component of the spheroid samples by using a short incubation period with a low concentration of Triton X-100 (0.1%). In comparison, because CUBIC [34] contains particularly high Triton X-100 levels (50%) to maximize lipid removal, its use leads to extensive protein loss, which lowers epitope concentrations and tends to reduce immunostaining [25]. 

To observe dynamic changes in spheroids at predetermined time points over a 10-day period, we matched the refractive indexes of the spheroid components, using a common and low-cost clearing reagent (85% glycerin in DDW). By simple immersion in a high refractive index hyperosmotic solution, dehydrated samples become adequately cleared [35]. In our assay, the spheroids became sufficiently transparent almost immediately and did not require prolonged incubation in the clearing agent before visualization with the confocal microscope. As a result, the preparation time for the samples, including staining, compared with other protocols for tissue clearing, were shortened from a couple of days to a few hours. Diluted glycerin showed an effective clearing capability and an ease of handling, by reducing the solution viscosity and formation of bubbles. 

Pancreatic adenocarcinoma spheroids were shown to recapitulate in a tissue-like manner, many tissue parameters related to cell interactions and organization [13]. Among the different pancreatic cancer cell lines examined in this paper, BxPC-3 cells formed the most compact and uniform spheroids within 24 h. BxPC-3 spheroid features were consistently dense, correlating with reports of pancreatic adenocarcinoma tumors in vivo, showing very limited optic imaging in a bright field. When labeling the spheroids with the lipid dye DiD (Ex: 644, Em: 665 nm), very low penetration was observed, with only the outer superficial cell layers of the spheroid stained. Interestingly, the commonly used lipid fluorescent molecule, Coumarin-6 (logP 6.9), was found to be an excellent cell membrane label and showed a high penetration capacity even though it has a peak excitation and emission in a shorter wavelength (Ex: 443 nm, Em: 494 nm). This might be further explored for wider cell labeling applications.

In all the experiments, spheroids that underwent our clearing showed a substantially better optical depth and image quality compared with uncleared samples, even when laser intensity and gain settings were enhanced in the uncleared samples, as shown with BxPC-3 spheroids (Figure 2). Cleared spheroids were visualized in detail up to ~200-microns, while uncleared sample images were limited to a ~50-micron depth.

The high heterogeneity in cancer, even from the same tumor origin, can be seen histologically and clinically. We examined whether cell origin, even from the same tumor type, affects spheroid assembly and imaging quality. Our previously published study showed that different cancer cell types dictate distinct unique spheroid morphology and density due to different spatial cell–cell interactions [13]. We utilized our clearing method when we compared 3 different types of pancreatic cancer cell lines, AsPC-1, BxPC-3, and PANC-1. We found that the 2 cell lines BxPC-3 and PANC-1, extracted from primary origin tumors, formed similar round-like spheroids, while AsPC-1, which is a metastatic cell line, formed grape-like spheroids. This is in agreement with previous reports by others, showing that colorectal cancer cell lines derived from metastatic origins also formed similar grape-like 3D tumors in laminin-rich-extracellular matrix [36]. Further research is needed to indicate whether there is a link between morphological appearances of pancreatic cell spheroids and their malignant potential. 

In a recent study of ours, we demonstrated how hypoxia and necrosis play a critical role in the progression of disease and tumor response to treatment [37]. Thus, detecting inner structures of large tissues and spheroids can provide valuable biological information shedding light on physiological processes at the tissue level. For this, BxPC-3 spheroids were imaged, using our clearing protocol and standard equipment at predetermined time points over prolonged growth periods. Our assumption was that due to limited access to the medium and oxygen in spheroids larger than 200-microns, the periphery cell layers would remain viable and proliferate while the core became hypoxic and eventually necrotic. Indeed, monitoring spheroids at different time points showed the development of a hollow core in BxPC-3 spheroids which may be a result of necrosis, similar to the tissue state in poorly vascularized tumor tissue regions [38,39]. Alternatively, it may be a result of differentiation or developmental morphogenesis. This should be determined in future studies.

Tumors form a complex multi-cellular microenvironment that has a major role in tumorigenesis, invasion, and tumor cell proliferation. The enormous complexity of tumor cell interactions with stromal cells and structural hierarchy makes it practically impossible to recapitulate all of the tumor’s in vivo properties by using only cancer cell lines ex vivo. Including multiple tissue components and stroma cells improves the authenticity of the ex vivo system. We demonstrated that co-cultured hybrid spheroids comprised of 3 different cell types: pancreatic tumor cells (TCs), patient derived pancreatic cancer fibroblasts (fibro), and endothelial cells (ECs) can be successfully assembled. The different cell types can be detected by specific antibodies that provide information related to their spatial interactions. This is thought to be governed by the origin of the cancer cells. Cancer associated fibroblasts (CAFs) are known to have a major role in tumor development and progression by secreting growth factors, interleukins, and matrix metalloproteases (MMPs) [40]. Pancreatic tumor cells induce expression of insulin-like growth-factor-1 to support cancer cell growth upon contact with fibroblasts. CAFs enhance ECM remodeling, promote cancer cell invasion, and chemoresistance [41]. Generally, pancreatic adenocarcinoma tumors are considered to be hypo-vascular, with low angiogenesis; their existing blood vessels often appear to be condensed and non-functional [42,43,44]. Detecting the organization of endothelial cells interacting with pancreatic cancer cells might be an indication of the level of angiogenesis, as was suggested in our previous study—showing vessel-like structures versus aggregated endothelial islets in angiogenic and low angiogenic cell lines, respectively [13]. Interestingly, in all the cases of pancreatic cancer cell lines that we tested using our clearing protocol, BxPC-3, AsPC-1, and PANC-1 fibroblast cells showed a scarce presence even though they were cultured in a ratio of 3:1:1 TCs:fibro:ECs. This is not correlated with known desmoplastic clinical observations. This phenomenon, when fibroblast cells disappear from co-culture spheroids, has been observed in other studies [16,45] and may be explained by either a short incubation period (4 days), suboptimal culture conditions, or contact inhibition by the tumor cells [45]. Additionally, the use of patient-derived cancer fibroblasts with immortalized cancer cells might provide a proliferation advantage to the tumor cells in the provided medium. In BxPC-3 spheroids, the endothelial cells were shown to be localized around the hypoxic core, a phenomenon which might be explained by excretion of vascular endothelial growth factor (VEGF) and additional proangiogenic factors [46,47], which are the main drivers of angiogenesis. In a recent study, Lazzari et al. co-cultured and characterized spheroids comprised of PANC-1, HUVECs, and MRC-5 lung derived fibroblast cells using a different cell ratio (1:4:9, PANC-1: HUVEC: MRC-5) in a total of 500 cells. Their spheroids were stained and imaged on day 4 and 7. A significant number of endothelial cells were located in the core of the spheroid on day 4 of their experiment, correlating with our findings of PANC-1 co-cultured spheroids. On day 7 of their experiment, fibroblasts were not detected [16], as per our observation on day 4 of culture. 

Although it is difficult to compare spheroids to a full in vivo tumor architecture, histological specimens obtained from mice xenografts, revealed that BxPC-3, PANC-1, and AsPC-1 tumors formed vascular density to a similar extent [48], but interestingly their ECM showed distinct differences. For example, a study compared collagen staining of BxPC-3 and PANC-1 tumors extracted from mice, showed that BxPC-3 tumors were more differentiated than PANC-1 tumors displaying thick filament collagen I and IV bundles separating clusters of the tumor cells, whereas PANC-1 tumors showed a divergent collagen arrangement with thin fibers of collagen I and IV surrounding the single tumor cells [49]. This observed pattern may be in accordance with the ECM rich core we detected in the BxPC-3 spheroids.

Since pancreatic adenocarcinoma is considered one of the most challenging solid tumors, with respect to drug delivery, many studies suggest that remodeling the tissue ECM by using enzymes or other methods may enhance drug perfusion and thus increase the therapeutic effect. In this regard, our clearing technique provides an opportunity to detect interactions with particle-based drug vehicles. Both PANC-1 and BxPC-3 spheroids were used to assess particle penetration. Interestingly, as shown in Figure 6, BxPC-3, which featured a round and dense morphology, demonstrates markedly lower permeability with both bead sizes, 0.8 and 2.4 µm, compared with PANC-1 spheroids. Two imaging setups, one of the surface and the other of the deeper layers, provided distinct information regarding adherence of particles and their penetration, respectively. These results support recently reported observations that nanoparticles of 20–500 nm in diameter reached longer depths in PANC-1 spheroids in comparison to BxPC-3 spheroids [15]. Evidently, the extracellular matrix of BxPC-3 spheroids is denser than in PANC-1 spheroids, a possible outcome of differences in their ECM composition. Kim et al. performed comparative proteomic profiling and determined 178 up-regulated proteins in BxPC-3 cells, compared with PANC-1 cells. A substantial segment of these proteins were linked to tight junction and adherent junction pathways [50]. 

Overall interaction of the 0.8 µm beads with control AsPC-1 spheroids was practically negligible, while their interaction and accumulation in enzymatic treated spheroids increased significantly, suggesting a major role of the ECM in limiting particle diffusion. It is important to note that the major differences were in the outer layers of the spheroids, as demonstrated in an internal slice of the spheroids (Figure 7). In the high enzymatic concentration of 0.2 u/mL, there is a possible damage to the cell membrane that should be taken into account indicated by the fade out of membrane staining while cell nuclei are still in site. This suggests a more dominant effect on adhesion than on transcellular mobility, possibly due to the short incubation time treatment with a low dose of enzymes and the relatively large dimensions of the particles. It is probable that smaller particles in the sub-micron scale would show different patterns of penetration into the 3D cell aggregates.

Based on our observations, we predict that our spheroid-clearing method could provide important information related to the heterogenetic biodistribution of drugs and drug carriers, and provide a platform as an ex vivo cancer model for optimizing drug delivery in respect to specific effects of both tissue and vehicle properties. This too might aid in the future, to rationalize the design of targeted delivery systems with preferable pharmacokinetic parameters. Further expansion of our technique would be to culture pancreatic cancer cells generated from surgically resected neoplasms, that can differentiate and create the in vivo-like microanatomy of a specific cancer tumor [51,52]. Such a system may be used for basic research on pancreatic cancer biology or for patient-specific drug testing, which represents a new form of precision medicine. 

## 4. Materials and Methods 

### 4.1. Statement

All experiments and methods were performed in accordance with relevant guidelines and regulations. Human patient derived cancer associated fibroblasts were extracted from fresh pancreatic adenocarcinoma tumor tissues as approved by the Institutional Review Board (IRB)/Ethics (Helsinki) committee of the Hadassah Medical Center (#920051034, and 0628–14-HMO). An informed consent was obtained from all subjects, and all methods were carried out in accordance with the relevant guidelines and regulations of the approved IRB protocols.

### 4.2. Cell Culture

Human pancreatic cancer cell lines BxPC-3, PANC-1, and AsPC-1 were obtained from ATCC (Manassas, VA, USA). Human umbilical vein endothelial cells (HUVEC) were purchased from Lonza (Basel, Switzerland). All cells were characterized before use, mycoplasma free (using an EZ-PCR mycoplasma test kit (Biological Industries, Beit HaEmek, Israel)) and used for the experiments up to p15. All cells were maintained in a 10% fetal calf serum (FCS) medium with penicillin/streptomycin (P/S) and kept in a humidified incubator at 37 °C with 5% CO_2_. For PANC-1 cell lines, DMEM (Life Technologies, Carlsbad, CA, USA) was used, BxPC-3 and AsPC-1 cells were maintained in RPMI-1640 (Life Technologies). HUVECs were grown in PeproGrow endothelial cell basal medium, supplemented with PeproGrow-MacroV kit (ENDO-BM & GS-MacroV, PeproTech, Rocky Hill, NJ, USA). Patient-derived pancreatic cancer fibroblasts were isolated from pancreatic cancer tissue by mincing and digesting for 30 min at 37 °C with 5% CO_2_, while slowly stirring in DMEM/F12 medium (Life Technologies) supplemented with P/S and 0.14 Wunsch units/mL of LiberaseTM Research Grade (Roche Diagnostics, Basel, Switzerland). Digested tissue was filtered through a cell strainer, after which the filtrate was diluted with a stop reaction medium: DMEM/F12 supplemented with P/S and 15% FCS and centrifuged at 220 RCF for 5 min. This process was repeated twice. Epithelial and fibroblast cells were separated by the sedimentation technique as previously described [52]. Fibroblasts were plated and maintained in EMEM medium (Life Technologies) supplemented with 15% FCS, 1% sodium pyruvate, P/S, and HEPES. 

### 4.3. Spheroid Formation Using 3D Petri Dish Well Array 

Master 3D Petri dish 35-well arrays (Microtissues Inc., Providence, RI, USA) were used to create micro-wells made up of 2% UltraPureTM agarose hydrogel (Invitrogen, Carlsbad, CA, USA). Micro-wells were seeded with 10,000 cells/well. Cells used in this assay were PANC-1, BxPC-3, AsPC-1, HUVECs, and patient-derived pancreatic adenocarcinoma cancer fibroblast cells. After seeding, 1 mL of the appropriate medium was added, and templates were incubated at 37 °C with 5% CO_2_ for 24 h up to 10 days. For hybrid spheroid experiments, pancreatic cancer cells were co-cultured with HUVECs and patient-derived pancreatic cancer fibroblast cells in the ratios: 3:1:1, respectively. Spheroids were formed using the 3D Petri dish templates as previously detailed. Since HUVECs are more sensitive to their environment compared with the other cells used in these experiments, and require an enriched medium for their growth, we used their optimized medium, as previously mentioned, for the co-cultured experiments. Light microscopy images were taken using Nikon Eclipse TS100 light microscope, x4 lens. 

### 4.4. Fixation, Permeabilization, and Immunostaining of Co-Cultured Spheroids 

At predetermined time points from their seeding, spheroids were fixed in 4% PFA and left at room temperature (RT) on an orbital shaker for 1 h. Spheroids were then washed and left in phosphate-buffered saline (PBS) for 5 min, repeated 3 times. Next, spheroids were removed from their molds by gentle pipetting using trimmed tips and transferred into 1.5 mL mini test tubes to ensure complete exposure to solution in the next steps which were all performed at room temperature (RT) on an orbital shaker. Fixed samples were incubated for 15 min in 0.1% Triton X-100 for permeabilization and were then washed and left in PBS for 5 min, repeated 3 times. Next, co-cultured spheroids were incubated with a blocking medium containing 3% normal goat serum (Vector Laboratories, Burlingame, CA, USA) for 1 h. Blocking was removed and primary antibodies: Mouse anti-human fibroblasts monoclonal antibody (Chemicon, Temecula, CA, USA, CBL271,1:100) and conjugated anti-CD31 antibody (Abcam, Cambridge, UK, ab215912, [JC/70A] Alexa Fluor 647, 1:100) were added successively in the 3% normal goat serum for an overnight incubation at 4 °C on an orbital shaker. Spheroids were washed 3 times in PBS, as previously mentioned, and incubated with a secondary labeled antibody: H&L [Cy3^®^] (Abcam, ab97035, goat anti-mouse igG H&L pre-adsorbed, 1:100) in 3% normal goat serum for 1 h followed by PBS washing.

### 4.5. DAPI, Coumarin-6, and DiD Staining

Spheroids were fixed and permeabilized as described above. After a single wash with PBS, they were incubated with 0.1% w/v of DAPI solution for 30 min and subsequently with DiD Lipophilic Tracers membrane dye (Ex/Em 644/665 nm, Invitrogen) for 30 min in a 1:200 volume ratio at RT on an orbital shaker. Next, the staining solution was removed, and spheroids were incubated with Coumarin-6 (0.2 mg/mL) for 30 min. After a 5-min wash with PBS, repeated 3 times, they were stored in PBS at 4 °C until taken for imaging.

### 4.6. Sample Preparation for Imaging

Sample preparation and processing is illustrated in Figure 1. To prepare samples for imaging, we attached ~0.5 × 0.5 cm spherical pieces of modeling clay in each corner of a 60 × 22 × 0.18 mm cover glass. Spheroids suspended in PBS were gently placed in the center of the cover glass, using a pipette with a trimmed tip. Residual PBS was removed, and 50 µL of a solution comprised of 85% glycerin and 15% DDW was gently added on top of the spheroids. To immobilize the spheroids for imaging, an additional cover glass was placed precisely above the sample and gently pressed down to create a ~1 mm gap between the cover glasses. This step immobilized the spheroids by preventing their movement during imaging. Confocal microscopy (Nikon confocal A1R, Tokyo, Japan, ×20 objective) was used for imaging and Z section imaging was performed. 

### 4.7. Evaluating Interactions of Spheroids with Fluorescent Labeled Particles

BxPC-3 and PANC-1 spheroids were formed as detailed above. In order to assess the penetration and interactions of inert particles with spheroids, after 24 h, SPHEROTM fluorescent polystyrene purple microparticles (Spherotech, Lake Forest, IL, USA) of 0.8 µm or 2.4 µm 0.001% *w*/*v* were added and incubated with the spheroids for 24 h. Samples were washed 3 times in PBS and subsequently fixed and stained as previously mentioned. Statistical analysis of the projected images, which provide a 2D representation of the 3D images, was performed using the NIS Element AR Analysis software (Nikon confocal software). 

### 4.8. Examining the Role of Spheroid ECM on Fluorescent Labeled Particle Penetration

To further elucidate the role of tumor ECM on particle diffusion in 3D samples, AsPC-1 spheroids were formed as detailed above and after 48 h were treated with digestive enzymes by incubating the samples with DMEM medium containing P/S and 0.1 or 0.2 Wunsch units/mL of LiberaseTM research grade for 5 min at 37 °C and 5% CO_2_ prior to incubation with 0.8 µm of SPHEROTM fluorescent polystyrene purple microparticles 0.001% *w*/*v* for 24 h. Statistical analysis of the projected images was carried out as mentioned above. 

### 4.9. Data Analysis and Statistics

Unless otherwise specified, experiments were performed in 2–3 repeats and were considered as qualitative results (Figure 2, Figure 3, Figure 4 and Figure 5). Spheroid radiuses were measured using ImageJ software (freeware from the US National Institutes of Health (NIH) and the Laboratory for Optical and Computational Instrumentation (University of Wisconsin)). Statistical data was analyzed on GraphPad Prism 8 (www.graphpad.com, San Diego, CA, USA) and all experiments had at least 4 independent replicates. Analysis of particle penetration into spheroids was normalized to the average volume of each spheroid group (assuming a spherical entity). Graphs of analyzed images show means and standard error of the means. In order to assess statistical significance, a Mann Whitney non-parametric test was performed in experiments where there were 2 groups, and the sample number was 4 in each group. One-way ANOVA with post-hoc Tukey’s test for comparison between means was performed for experiments containing more than 2 groups and a total sample number of 15. Statistical significance is indicated on the respective graphs.

## 5. Conclusions

There is a constant search for novel ex vivo models that can provide information related to tissue dynamics and their microenvironment. The ability to extract information related to cell–cell interactions, their spatial organization in a tissue, and morphogenesis is critical in 3D cellular models. We concluded that establishing an optimized clearing procedure for spheroids—our chosen 3D cellular model—is possible, and that such a procedure provides the necessary details at the single cell level, within a given thick sample. Our clearing protocol using standard equipment can both save time and enable high-level imaging that will reveal important biological processes. We tested our protocol in studies of cancer morphology, tissue processes, and particle penetration in spheroids and extracted valuable information which was previously difficult to obtain. 

## Supplementary Materials

Supplementary materials can be found at https://www.mdpi.com/1422-0067/21/20/7703/s1.
