# Supplementary material for: Rapid Clearing for High Resolution 3D Imaging of Ex Vivo Pancreatic Cancer Spheroids
Source: Int J Mol Sci. 2020 Oct 18;21(20):7703. doi: 10.3390/ijms21207703 (PMC7589457; doi:10.3390/ijms21207703)
Supplement: Supplementary file 1 [file ijms-21-07703-s001.pdf]

Supplementary Materials

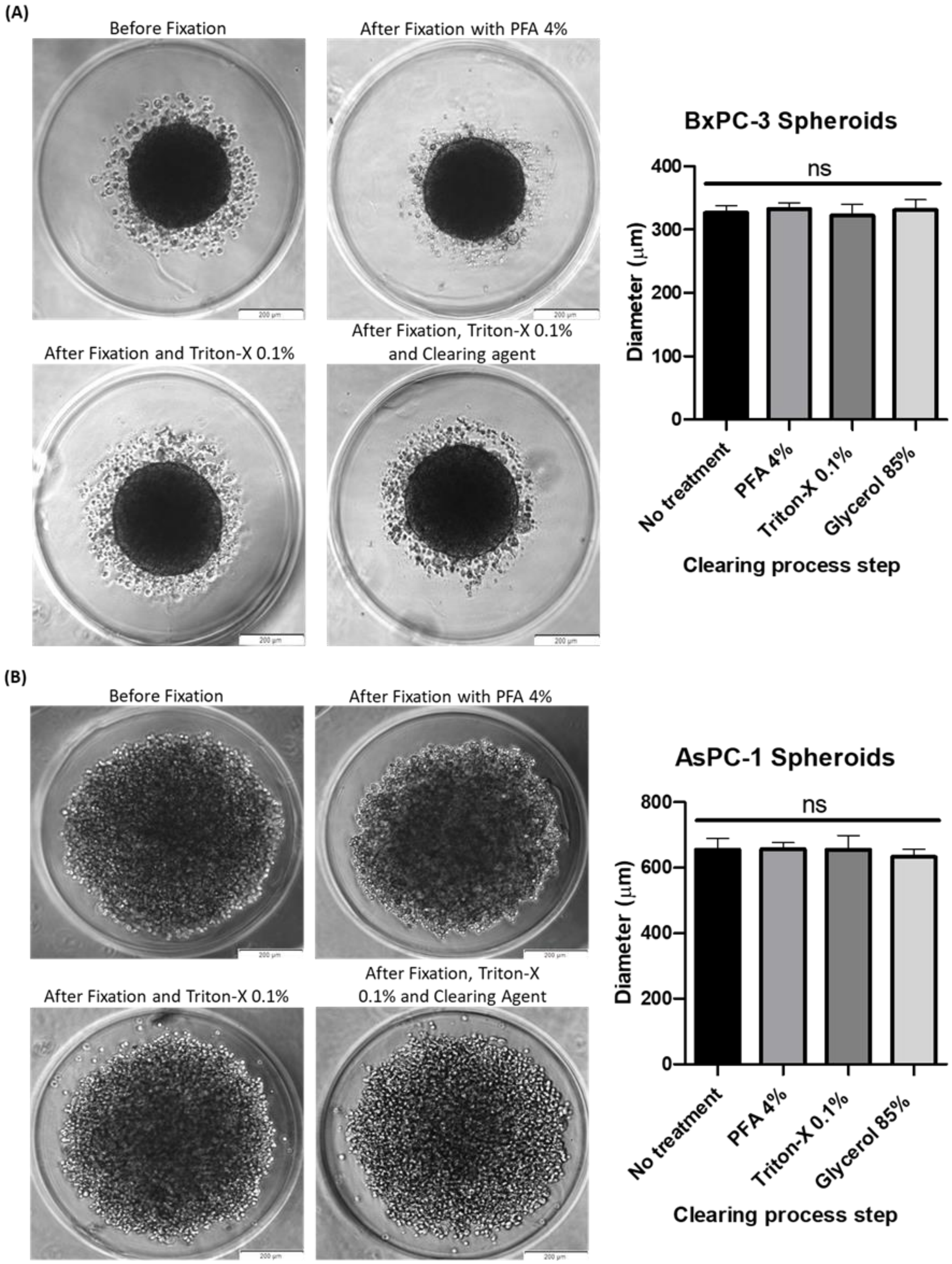

Figure S1. Comparing the size of BxPC-3 and AsPC-1 spheroids before and after each step of the clearing process. Light microscopy images of (**A**) BxPC-3 and (**B**) AsPC-1 spheroids taken before fixation, after fixation (with PFA 4%), following treatment with Triton-X 0.1% and after treatment with glycerin 85%. Results confirm the process had no effect on the spheroids' size. Radiuses were calculated using ImageJ and data was statistically analyzed using GraphPad Prism 8 (n=20-28).
